# DR services in Fiji: attitudes, barriers and screening practices

**Published:** 2015

**Authors:** Bridget Kool, Mai Webber, Judith McCool

**Affiliations:** Senior Lecturer: University of Auckland, Auckland, New Zealand. Email: b.kool@auckland.ac.nz; Postgraduate Student: Auckland, New Zealand.; Senior Lecturer: Auckland, New Zealand.

The aim of this study was to describe the attitudes and perceptions of primary health care doctors in Fiji regarding the importance of eye care in diabetes mellitus (DM) management, to explore current eye care practice, and to investigate awareness and use of relevant clinical practice guidelines. The study builds on earlier research conducted in Fiji that identified a rapid increase of late-stage DR patients presenting for treatment, at a time when surgery was the only option.^1^ A cross-sectional survey of primary care doctors, both private (general practitioners [GPs], n=21) and from the public health service (medical officers [MOs], n=10), involved in the management of patients with DM was conducted in 2013 in the Central Division of Fiji. The survey topics included: clinical experience and training relevant to the management of eye care among DM patents, current practice in relation to the care of these patients, and sources of relevant knowledge. Clinical vignettes (scenarios) were used to explore ‘usual practice’ for patients presenting with various stages of eye disease.

The results of the survey indicate that clinicians perceive ‘eye disease’ and/or DR as their least ‘most-significant concern’ after a patient has been diagnosed with DM. When asked to identify their most significant concerns after diagnosing a patient with DM, ‘Glucose control’ and ‘renal disease’ were the most commonly selected ‘most significant concern’ (n = 14) by GPs, and ‘Renal disease’ by MOs.

Clinicians felt that a general practice doctor is not the most appropriate individual to perform diagnostic testing/screening for DR in patients with DM. Just over half of all GPs, and most (8/10) MOs, ‘agreed’ that if a patient with DM effectively manages their glucose levels, they will not develop DR. (Although good glucose and blood pressure management can reduce the risk of developing DR, it does not eliminate it. All patients with diabetes should be screened for DR every year.)

Over three-quarters of clinicians responded that DR screening is ‘always’ performed on diagnosis of DM. All of the GPs surveyed indicated they would ‘always’ refer a patient for DR screening on signs of decreased visual acuity (VA). One MO indicated that they ‘never’ screen for DR even with decreased VA, and the remainder indicated they would ‘almost always’ or ‘always’ refer the patient for DR screening. The majority of GPs (15/21) and MOs (7/10) indicated that they ‘sometimes’ conduct eye examinations themselves whilst the remaining clinicians indicated that they ‘never’ conduct the examinations themselves.

Interestingly, most GPs (16/21) and all MOs felt that patients failed to seek specialist eye care because of a ‘lack of awareness’, more so than any other factor. A small minority of MOs thought their patients were ‘worried about the consequences’ of being diagnosed with a chronic illness. Respondents were asked to identify what other issues were causing patients to delay seeking specialist eye care. Three GPs indicated that patients lacked funds and support from home to pursue treatment options, and one indicated that there was a perception that it was simply a ‘feature of ageing’. One GP indicated that patients often feared qualified people and thought that they would not be compassionate or able to relate to their concerns.

Over half (13/21) of GPs and almost three-quarters of MOs (7/10) surveyed indicated that they used clinical guidelines to guide their management of patients with DM. Most GPs (10/13) and MOs (6/7) felt that the guidelines they utilise ‘require revision’. Almost half (8/20) of all respondents who use guidelines stated they used the standard Fijian Ministry of Health guidelines for the management of DM, with a small number reporting using the World Health Organisation (WHO) guidelines on DM Management and the Australian Ministry of Health guidelines. Previous research indicates that, if clinicians consider guidelines to be ‘burdensome’, then they are less likely to use them.^2^,^3^

Responses to clinical vignettes for the management of DM suggest that the majority of clinicians adhere to best practice. Key areas of variation were in relation to the management of DR in the context of pregnancy and DR screening on initial diagnosis of DM. Vignette two examined clinicians' responses when confronted with a patient recently diagnosed with DM. The results of this vignette indicated that only half of clinicians would immediately refer this patient for DR screening, the remaining clinicians would only refer this patient for retinal screening if they developed visual symptoms or if their DM progressed beyond its current state. Vignette four assessed clinicians' responses when managing a pregnant woman with DM. The results indicated that, contrary to best practice, a proportion (6/31) of clinicians were ‘not concerned at all’. Clinicians who do not use guidelines were approximately twice as likely to be ‘not concerned at all,’ compared with those who reported using guidelines.

Clinicians indicated that a lack of training and/or the availability of workshops was a barrier to their ability to effectively manage DR. Basic eye health screening tools, such as ophthalmoscopes, and more sensitive equipment such as colour digital fundus imaging were notably lacking in the practice settings of those who participated in this study. The participants indicated a desire to receive more training in DM eye care. Updating clinician skills at the primary care level has been shown to improve rates of diagnosis and reduce burden on secondary care facilities.^4^

## Discussion

Responses from clinicians in this survey suggest they do not see DR as a significant complication of DM that has the potential to affect patient outcomes and subsequent quality of life. Feeling under-resourced in terms of staff, facilities, supplies, and ultimately time to deal with DM and its complications in a comprehensive manner was a common sentiment from clinicians. Due to a combination of financial reasons, lack of knowledge, apathy, and/or a reluctance to alter lifestyle, clinicians felt that patients in Fiji delay seeking medical attention for DM and are failing to comply with clinical treatment protocols. Finally, issues with communication between agencies and providers were a common theme, with several MOs indicating they were unaware of what occurred after referring their patients to a specialist.

The findings of this study suggest that several barriers exist which hinder the optimal treatment or management of DR.

- A belief by practitioners that DR is a less significant problem than other complications of DM.
- The fact that clinicians' knowledge of disease progression, and diagnostic techniques, were under-developed.
- Clinicians' ambivalence towards DM management, and by proxy, their lesser focus on DR.

The ambivalence towards DM management and lesser focus on DR may be attributed to a range of factors, including: persistent poor patient compliance with treatment advice, the limited time that clinicians can afford to spend with patients, and their willingness to refer to specialist services which are available in Fiji. An important factor to consider is the appropropriateness of primary care doctors diagnosing DR without the appropriate diagnostic tools, such as slit-lamp biomicroscopy and/or stereo fundus photography.

The study needs to be considered in light of some limitations. The clinicians surveyed are not representative of all clinicians in Fiji; respondents were self-selected which may have resulted in some selection bias;^5^ and the information collected relied on self-reporting and was unable to be verified. All of these factors have the potential to introduce bias to the study. However, despite these limitations, the findings are likely to be relevant to clinicians and other eye health care workers practicing in low-resourced settings as they provide useful insights into the challenges eye care providers face both from delivery of care and workforce development perspectives.

Research that explores specific motivating or demotivating factors affecting clinicians in Fiji (and in other low-resourced settings) and other eye health care workers accessing and applying clinical guidelines in their day-to-day practice is required, as dissemination of guidelines alone does not appear to alter management of DR.^6^ Analysis of routinely collected data such as that maintained by specialist eye clinics is required to ascertain the duration between diagnosis of DM and initial DR screening and/or treatment. This information would provide a reference point for assessing the efficacy of future DR screening/management programmes in Fiji and other low-resourced settings.
